# The Pain Crisis: What It Is and What Can Be Done

**DOI:** 10.1155/2012/703947

**Published:** 2012-09-19

**Authors:** Barry J. Sessle

**Affiliations:** Faculties of Dentistry and Medicine, University of Toronto, 124 Edward Street, Toronto, ON, Canada M5G 1G6

## Abstract

Chronic pain is present in epidemic proportions in most countries, is often unrelieved, and has a huge socioeconomic impact. It is not just a “medical” illness but indeed is a problem that faces all healthcare professional fields. Several steps are identified to address this crisis. These include approaches to enhance pain awareness and access to timely and effective care for pain, and educational and research approaches to improve the knowledge base of healthcare professionals and students and diagnostic and management procedures for pain. Several opportunities to enhance pain understanding, access, and management are also identified.

## 1. Introduction

Despite recent advances in our understanding, diagnosis, and management of pain, several problems confront the pain field, especially in the case of chronic pain which remains a public health problem of epidemic proportion in most countries. This is largely due to the limited levels of (a) awareness of pain, particularly its socioeconomic impact and burden, and access to timely and appropriate care, (b) pain education, especially of health professionals, and (c) research into pain mechanisms and into ways to improve diagnostic and management approaches and healthcare delivery. This pain crisis has been highlighted over the past 2 decades by several authors and again recently by the recent report in the USA by the Institute of Medicine (IOM) [1–7]. This article will focus on these areas and provide for each some possible means to address the current crisis of chronic pain.

## 2. Awareness of Pain and Its Impact

Pain is a subjective individual experience encompassing sensory, cognitive, emotional, and social dimensions. One person may report a moderate level of pain following an injury while another person with a similar injury may report a much higher, or lower, pain level. This individual-by-individual experience of pain depends on numerous features that include each person's unique genetic features and cognitive, motivational, emotional, and psychological state as well as environmental factors stemming from their gender, past experiences and memories of pain, cultural and social influences, plus their general health condition.

Pain does serve an important vital function as a warning signal of tissue damage, resulting from an accidental trauma, infection, or inflammation, for example. It usually disappears after the injured tissue has healed. In contrast to such acute pain episodes, chronic pain is usually considered as having no biological role but is associated with changes in the peripheral and central nervous systems that contribute to its persistence. Because of these changes that involve alterations in brain morphology, physiology, and neurochemistry [8–10], chronic pain is now often viewed as a neurological disorder akin to other chronic medical illnesses and conditions involving analogous alterations in the nervous system (e.g., epilepsy, Parkinson's disease); in other words, chronic pain is a disease or illness in its own right. Furthermore, chronic pain may be a pain disorder per se (e.g., fibromyalgia, trigeminal neuralgia, temporomandibular disorders, migraine) although affecting other functions (e.g., mobility, cognitive function), or be an accompaniment of many chronic diseases and disorders (e.g., arthritis, diabetes, cancer, HIV/AIDS). It can also result from acute pain since approximately 20% of acute pain conditions can transition into chronic pain, especially if the acute pain is not appropriately managed [11].

Chronic pain is very prevalent, with estimates ranging from 12–30% depending on the country surveyed [1, 4, 12–14]. Yet, it is a “silent epidemic” as several studies have noted, since there is little awareness of its prevalence and social and economic costs for the patient and society as a whole [2, 4, 6, 7, 15–18]. These socioeconomic consequences include reduced quality of life, negative impact on relationships, job loss or reduced job responsibilities, ineffective management of pain, and increased rates of depression. In addition to the personal social and psychological “costs” for the person suffering with chronic pain, there are also considerable economic costs to the patient and to society as a whole. For example, in Canada, the personal financial costs for pain patients is close to $1,500 per month and the direct and indirect costs to the Canadian economy have been estimated to be >$30 B/year. The recent IOM report places this cost at >$500 B/year in the USA; this economic burden is higher than the healthcare costs for heart disease, cancer and diabetes combined, and stems from the costs of healthcare services, insurance, welfare benefits, lost productivity, and lost tax revenues, among others [2, 5, 16, 18].

A pain crisis exists, and it is relatively unrecognized by the public and policy makers. Plus, it is not going to get any better unless concerted efforts are made to enhance awareness of pain and its huge socioeconomic impact since demographic research suggests that chronic pain conditions will become even more of a health problem and socioeconomic burden [18–21]. Over the coming years, changing demographics will result in a higher proportion of the population of most countries being middle-aged and elderly, the age cohorts where most chronic pain conditions are particularly evident and usage of the healthcare system is particularly high.

The pain crisis is compounded, even in so-called developed countries, by the difficulty that many patients in pain, especially chronic pain, have in gaining timely access to appropriate pain care in spite of such access being a basic human right recognized by the United Nations, World Health Organization, and IASP [11, 22–25]. Timely access is essential since chronic pain patients experience considerable deterioration in their psychological well-being and health-related quality of life while they wait until treatment can be instituted; the longer they have to wait for relief of their pain, the more severe the impact, the greater the degree of chronicity, and the larger the cost to the healthcare system [11].

Because of the cognitive, emotional, and psychological effects that may be associated with pain, a biopsychosocial concept of pain has emerged over the past 2-3 decades along with considerable evidence supporting management approaches addressing the psychosocial aspects of a patient with chronic pain [2, 3, 5, 22]. Yet such approaches, and even management strategies based on pharmacological, surgical or other interventions, are difficult for many patients to access. This is because of several barriers that reflect the nature of organizational, structural, educational, and reimbursement features of current healthcare systems in most countries. For example, most patients in pain first try to seek care from primary healthcare professionals, and indeed pain complaints may account for 40% or more of patient visits to physicians, for example [2, 26–28]. However, as pointed out in the recent IOM Report [2], primary care in the USA usually is organized and reimbursed in such a manner that precludes comprehensive patient assessment, to the pain patient's detriment. Insurance coverage may favour some procedures (e.g., drug interventions, surgery) over behavioural or physical therapies that in many cases may be more beneficial to the patient.

Access to appropriate care in a timely fashion is a problem particularly in most developing countries. For example, access to opiate drugs is very limited because of factors such as costs, opiate phobia, government restrictions, inability to access prescribing clinicians, and in some countries the infrastructure of the healthcare system is insufficient for pain patients to obtain care, even for those with horrific injuries [3, 22, 29, 30]. And even in developed countries, some of these features are also evident, compounded by limited evidence-based data on treatment outcomes and by abuse or misuse by a minor segment of society of some drugs used for pain patients. The possibility of such abuse or misuse runs the risk of legislation being put in place that negatively affects access by legitimate pain patients to appropriate analgesic medications. It could also compromise chronic pain management, resulting in undertreatment and even pseudo-addiction.

Several steps can, and should, be taken to address the pain crisis, especially from the point of view of awareness and access to care. As the recent IOM report [2] has noted, it necessitates a “cultural” shift in the way clinicians and the public view pain and its treatment. Policy makers should also be included in this cultural realignment. There needs to be increased efforts to

- inform the public, and government and policy makers about pain and its socioeconomic impact and problems with access to timely, appropriate pain management,
- develop integrated, comprehensive strategies for pain prevention, management, education, and research that will result in enhanced levels of access and care for pain patients,
- develop and widely distribute pain information sheets and articles for patients, healthcare professionals, government/policy makers, and so forth,
- inform and collaborate with other stakeholders in these initiatives, such as patient advocacy groups which would include educational material on pain self-management and prevention so that pain patients play a more active role in dealing with their pain,
- ensure a sufficient number of accessible pain clinics are available so that timely and appropriate multidisciplinary care is available to all citizens,
- enhance collaborative activities between primary healthcare providers and pain specialists that include referrals to such multidisciplinary pain management clinics,
- ensure pain management/medicine becomes a recognized and popular healthcare specialty,
- advocate to government/policy makers, insurance companies, and so forth, so that evidence-based management approaches are widely available, and
- develop guidelines for appropriate wait times for access to pain care.

In recent years, several steps have taken to address these points. They include the establishment by the International Association for the Study of Pain (IASP) of the Global Year Against Pain, an initiative linked to national and regional awareness initiatives in IASP chapters around the world. Another IASP initiative has been the international Pain Summit (held in Montreal following the IASP World Congress on Pain in September 2010) and this has been complemented by national pain summits. Another analogous approach is the recent European-Union-sponsored Symposium [31] calling on all European governments to take action to address the huge societal impact of pain. Pain advocacy groups have also held events for policy makers both locally and nationally to raise their pain awareness. Linked to some of these initiatives have been steps to encourage policy makers to develop and put in place a comprehensive pain strategy to address the many facets of the pain crisis. In the case of access to care, initiatives already taken here include a recent IASP international Task Force, cochaired by Mary Lynch and myself, which developed guidelines to address the timely and appropriate management of chronic pain on a global basis. These guidelines are based on the principle that all people have the right for timely access to appropriate care for chronic pain and that each nation should take steps to ensure that the principle is applied to all its citizens. Other steps taken in some countries have been the establishment of procedures for accreditation of hospitals and other healthcare organizations that has taken into account enhancement of access to appropriate pain management as well as of the quality of that care. In Canada, steps have been taken in some provinces to improve access at the community level and to advocate successfully for the establishment of a pain specialty [32–34].

## 3. Education of Health Professionals and Students

In addition to the public and policy makers being an advocacy target in order to “educate” them about pain and its socioeconomic burden, another target must be healthcare professionals and students in health professional programmes. Several recent reports have noted the inadequate knowledge of most healthcare professionals about pain and its diagnosis and management and how this impacts on their decision making [2, 3, 5, 6, 35–39]. As a result, costly or inappropriate or inadequate procedures are often carried out when other approaches could be more appropriate (e.g., counseling, prevention, self-management). There is still variability amongst clinicians in applying new and even existing knowledge about pain and its management. This is reflected in the documentation of inappropriate or indeed lack of treatment for patients with cancer, HIV/AIDS, neonatal, and postoperative pain, among others [35, 40–44]. This stems from inadequate knowledge or outdated attitudes about pain diagnosis or management. However, it may be compounded by several factors including the limited availability of effective analgesics and other pain-relieving approaches in many countries, limited access to pain treatment (as noted above), and use of management approaches that have not been validated or fully tested for their sensitivity and specificity. The variability in applying new knowledge and standards of practice and management of pain may be particularly evident in regions with economic and infrastructure limitations [29].

Major factors contributing to the misunderstanding and limited knowledge of pain by many healthcare professionals include the difficulty of treating most chronic pain conditions and the numerous other “competing” diseases and illnesses that most practicing clinicians have to be aware of and competent to manage. Another factor is their relatively poor understanding of chronic pain mechanisms because of the limited pain education that the vast majority of clinicians receive in their undergraduate and postgraduate professional programmes. At most health professional programmes, the topic of pain occupies only a minor component of the curriculum. This is evident in surveys carried out in North America [39] and Europe [45] where, for example, dental and medical students receive on average only 15-16 hours of formal education about pain throughout their multiyear programme; yet veterinary medicine is far ahead of the other professional programmes. Such neglect of pain in the vast majority of health professional programmes is incongruous given (i) the current high prevalence of pain and its socioeconomic costs, (ii) the changing demographics that suggest future increases in the incidence of chronic pain, (iii) that pain is an integral component of practice in medicine, dentistry, nursing, pharmacy, and other health disciplines, and (iv) that pain is indeed one of the major reasons for patients visiting physicians, dentists, and other healthcare professionals. The relative neglect of pain in the curriculum is also evident from the competency and accreditation requirements of graduating healthcare professionals and their educational programmes. For example, my own discipline (dentistry), only 2 of 47 and 2 of 39 competency requirements, respectively, in Canadian and U.S. dental schools (Association of Canadian Faculties of Dentistry; American Dental Education Association) relate to pain and its diagnosis and management; competency requirements in medicine also play little attention to pain.

Several steps are needed to address this imbalance and improve the understanding of pain and its management by clinicians, for the benefit of the patient in pain. They include

- increase pain curricular time in all health professional programmes,
- utilize current pain curricula developed by national and international pain-related organizations,
- ensure pain is taught within a biopsychosocial framework and in an integrated interdisciplinary manner that reflects its multidimensional nature,
- ensure there is sufficient coverage of pain in accreditation requirements of health professional programmes and in practice standards for healthcare professionals, hospitals, and other healthcare facilities,
- synthesize new pain-related information for widespread readership by healthcare professionals, and
- ensure effective knowledge transfer and application about pain and its management.

These various approaches are essential to improve the healthcare professional's knowledge about pain, although it is recognized that local academic constraints and school “politics” likely will make it difficult to increase curricular content on pain in many healthcare professional programmes. But they can be accomplished, as evidenced by an initiative at my own university, the University of Toronto, where the shortcomings in the curricula for medical, dental, nursing, pharmacy, and other health professional students were recognized. As a result, an interfaculty pain curriculum dealing with the many facets of pain, from basic science to clinical management to patient issues, was put in place 10 years ago for these students; it has continued to have successful outcomes [38, 46, 47]. Steps are being taken to address this problem in other countries, including the USA [48, 49].

## 4. Pain Research

There have been significant advances in our understanding of pain and improvements in pain management approaches (see Table 1), and more exciting advances can be expected in the coming decades as research developments in brain imaging, biomarkers, genetics, behavioural strategies, and so forth, are applied to pain diagnosis and management. Nonetheless, the evidence base for some of these approaches is limited, and in addition further research is needed to clarify the mechanisms, aetiology, and pathogenesis of most chronic pain conditions. This includes research directed at the mechanisms accounting for the differences between individuals in their pain experience, at the mechanisms and factors involved in the transition from acute to chronic pain, at more clinically applicable animal models of pain, and at translational approaches linking experimental pain findings with improved pain management in clinical settings. Also needed is more research addressing the basic science and clinical utility of recent technologies utilizing brain imaging, biomarkers, genotyping, and so forth, so as to identify those patients who will most benefit from newly developed therapeutic approaches for pain. There also needs to be an increased research focus on the multitude of current approaches used to manage pain which for many lack an appropriate evidence basis [2, 3, 5, 20, 50].

A critical factor essential for underpinning such multifaceted research is more research funding directed at studies into pain mechanisms, diagnosis, and management. Pain research funding has always been hugely out of proportion to the prevalence and socioeconomic impact of pain, compared to other less common conditions (e.g., cancer, HIV/AIDS, heart disease, arthritis, epilepsy). Recent surveys in the USA and Canada, for example, reveal the stark reality that funding for pain research is <1% of the research budgets of the US National Institutes of Health and the Canadian Institutes of Health Research [51, 52]. Such limited funding not only hampers the timely advancement of the understanding of pain and improvements in diagnostic and management approaches but also limits the number of scientists and clinicians attracted to pain research.

To address this aspect of the pain crisis, a number of steps should be taken. These include

- raising awareness of policy makers and funding agencies of the need to place much more emphasis on pain research by increasing opportunities for training basic science and clinical pain researchers and by increasing pain research funding,
- ensuring established and potential pain researchers are aware that the pain field can be more rapidly advanced by utilizing recently developed and emerging technologies (e.g., in molecular biology, genetics, brain imaging),
- increasing emphasis on inter-/multidisciplinary and translational research,
- developing more expeditious approaches to assess and apply new therapies for pain,
- applying new basic science knowledge and evidence-based principles in clinical pain research, and
- developing more substantive clinical data bases and placing greater emphasis placed on epidemiological studies and randomized clinical trials.

## 5. Conclusion

Chronic pain is in epidemic proportions in most countries. It carries with it huge socioeconomic burdens and it is often unrelieved. The last 4 decades have seen some remarkable advances in our understanding of pain mechanisms and improvements in pain diagnosis and management, and healthcare delivery in general. Nonetheless, considerable gaps in knowledge and approaches still exist. There is need to enhance pain awareness and education and ensure timely access to appropriate pain care, and to enhance pain research activity and resources. Several approaches have been identified to address this pain crisis. Since there is considerable variability between countries in their healthcare policies, programmes, resources, and educational programmes, many of the approaches and strategies outlined above will have to be customized to each country's socioeconomic, educational, healthcare delivery, and research infrastructures.

## Figures and Tables

**Table 1:** Recent advances in pain research and management.

(i) Identification of peripheral and central nociceptive processes involving nonneural as well as neural mechanisms	
(ii) Discovery of several endogenous neurochemicals and intrinsic pathways in the brain and their influences on nociceptive	
transmission and behaviour	
(iii) Development of concepts and insights of the neuroplasticity of pain processing that can lead to chronic pain	
(iv) Rapid advances in the fields of brain imaging, biomarkers, genetics, and molecular biology as well as their applicability to the pain field	
(v) Recognition of the multidimensionality of pain and importance of biopsychosocial factors in pain expression and behaviour	
(vi) Improvements in surgical, pharmacological, and behavioural management of pain:	
(a) more effective and varied drug-delivery systems	
(b) broader range of analgesics and other drugs for management of pain and related conditions	
(c) spinal cord and brain stimulation, transcutaneous electrical nerve stimulation	
(d) physical/rehabilitative medicine	
(e) new or improved surgical approaches	
(f) cognitive behavioural therapy	

## References

[1] Breivik H, Collett B, Ventafridda V, Cohen R, Gallacher D (2006). Survey of chronic pain in Europe: prevalence, impact on daily life, and treatment. *European Journal of Pain*.

[2] Institute of Medicine (2011). Relieving pain in America: a blueprint for transforming prevention, care, education, and research. *Report Brief*.

[3] Loeser JD (2012). Five crises in pain management. *Pain: Clinical Updates*.

[4] Schopflocher D, Taenzer P, Jovey R (2011). The prevalence of chronic pain in Canada. *Pain Research & Management*.

[5] Sessle BJ, Dostrovsky JO, Carr DB, Koltzenburg M Outgoing president’s address: issues and initiatives in pain education, communication, and research.

[6] Sessle BJ (2011). Unrelieved pain: a crisis. *Pain Research & Management*.

[7] Stewart WF, Ricci JA, Chee E, Morganstein D, Lipton R (2003). Lost productive time and cost due to common pain conditions in the US workforce. *Journal of the American Medical Association*.

[8] May A (2008). Chronic pain may change the structure of the brain. *Pain*.

[9] Salter MW (2004). Cellular neuroplasticity mechanisms mediating pain persistence. *Journal of Orofacial Pain*.

[10] Sessle BJ (2000). Acute and chronic craniofacial pain: brainstem mechanisms of nociceptive transmission and neuroplasticity, and their clinical correlates. *Critical Reviews in Oral Biology and Medicine*.

[11] Lynch ME, Campbell F, Clark AJ (2008). A systematic review of the effect of waiting for treatment for chronic pain. *Pain*.

[12] Boulanger A, Clark AJ, Squire P, Cui E, Horbay GLA (2007). Chronic pain in Canada: have we improved our management of chronic noncancer pain?. *Pain Research and Management*.

[13] Moulin DE, Clark AJ, Speechley M, Morley-Forster PK (2002). Chronic pain in Canada—prevalence, treatment, impact and the role of opioid analgesia. *Pain Research and Management*.

[14] Reitsma ML, Tranmer JE, Buchanan DM, VanDenKerkhof EG (2012). The epidemiology of chronic pain in Canadian men and women between 1994 and 2007, results from the longitudinal component of the national population health survey. *Pain Research & Management*.

[15] Henry JL (2008). The need for knowledge translation in chronic pain. *Pain Research & Management*.

[16] Koestler AJ, Myers A (2002). *Understanding Chronic Pain*.

[17] Lee P (1994). The economic impact of musculoskeletal disorders. *Quality of Life Research*.

[18] Philips CJ, Schopflocher D, Taenzer P, Rashiq S, Schopflocher D (2008). The economics of pain. *Health Policy Perspectives on Chronic Pain*.

[19] Farrell MJ (2000). Pain and aging. *American Pain Society Bulletin*.

[20] Green CR (2008). The healthcare bubble through the lens of pain research, practice, and policy: advice for the new president and congress. *Journal of Pain*.

[21] Helme R, Gibson S, Crombie IK (1999). Pain in older people. *Epidemiology of Pain*.

[22] Bond M, Breivik H (2004). Why pain control matters in a world full of killer diseases. *Pain: Clinical Updates*.

[23] Brennan F, Cousins MJ (2004). Pain relief as a human right. *Pain: Clinical Updates*.

[24] Cousins MJ, Lynch ME (2011). The Declaration Montreal: access to pain management is a fundamental human right. *Pain*.

[25] Peng P, Choiniere M, Dion D (2007). Challenges in accessing multidisciplinary pain treatment facilities in Canada. *Canadian Journal of Anesthesia*.

[26] Mäntyselkä P, Kumpusalo E, Ahonen R (2001). Pain as a reason to visit the doctor: a study in Finnish primary health care. *Pain*.

[27] McCarberg BH (2011). Pain management in primary care: strategies to mitigate opioid misuse, abuse, and diversion. *Postgraduate Medicine*.

[28] Upshur CC, Luckmann RS, Savageau JA (2006). Primary care provider concerns about management of chronic pain in community clinic populations. *Journal of General Internal Medicine*.

[29] Bond M (2011). Pain education issues in developing countries and responses to them by the International Association for the Study of Pain. *Pain Research & Management*.

[30] Williams AC (2009). Chronic pain in survivors of torture. *Pain: Clinical Updates*.

[31] Varrassi G (2011). Symposium on the Societal Impact of Pain 2011: European Commissioner John Dalli calls for a better understanding of ‘pain’in Europe. *Pain Management*.

[32] Bouchard P, Rashiq S, Schopflocher D, Taenzer P, Jonsson E (2008). The Quebec experience. *Chronic Pain: A Health Policy Perspective*.

[33] Taenzer P (2008). Chronic non-cancer pain in Canada: a public health perspective. *Report To Public Health Agency of Canada*.

[34] Royal College of Physicians

[35] Breitbart W, Jensen TS, Turner JA, Wiesenfeld-Hallin Z Pain in AIDS.

[36] Green CR, Wheeler JRC, LaPorte F (2003). Clinical decision making in pain management: contributions of physician and patient characteristics to variations in practice. *Journal of Pain*.

[37] Pizzo PA, Clark NM (2012). Alleviating suffering 101—pain relief in the United States. *The New England Journal of Medicine*.

[38] Watt-Watson J, Hunter J, Pennefather P (2004). An integrated undergraduate pain curriculum, based on IASP curricula, for six Health Science Faculties. *Pain*.

[39] Watt-Watson J, McGillion M, Hunter J (2009). A survey of prelicensure pain curricula in health science faculties in Canadian universities. *Pain Research and Management*.

[40] Demyttenaere S, Finley GA, Johnston CC, McGrath PJ (2001). Pain treatment thresholds in children after major surgery. *Clinical Journal of Pain*.

[41] Furlan AD, Reardon R, Weppler C (2010). Opioids for chronic noncancer pain: a new Canadian practice guideline. *Canadian Medical Association Journal*.

[42] Gilson AM, Joranson DE, Maurer MA (2007). Improving state pain policies: recent progress and continuing opportunities. *CA Cancer Journal for Clinicians*.

[43] Stalnikowicz R, Mahamid R, Kaspi S, Brezis M (2005). Undertreatment of acute pain in the emergency department: a challenge. *International Journal for Quality in Health Care*.

[44] Watt-Watson J, Stevens B, Garfinkel P, Streiner D, Gallop R (2001). Relationship between nurses’ pain knowledge and pain management outcomes for their postoperative cardiac patients. *Journal of Advanced Nursing*.

[45] Briggs EV, Carr ECJ, Whittaker MS (2011). Survey of undergraduate pain curricula for healthcare professionals in the United Kingdom. *European Journal of Pain*.

[46] Hunter J, Watt-Watson J, McGillion M (2008). An interfaculty pain curriculum: lessons learned from six years experience. *Pain*.

[47] Watt-Watson J (2011). Moving the pain education agenda forward: innovative models. *Pain Research & Management*.

[48] Murinson BB, Nenortas E, Mayer RS (2011). A new program in pain medicine for medical students: integrating core curriculum knowledge with emotional and reflective development. *Pain Medicine*.

[49] Vadivelu N, Kombo N, Hines RL (2009). The urgent need for pain management training. *Academic Medicine*.

[50] Chou R, Ballantyne JC, Fanciullo GJ, Fine PG, Miaskowski C (2009). Research gaps on use of opioids for chronic noncancer pain: findings from a review of the evidence for an American pain society and American academy of pain medicine clinical practice guideline. *Journal of Pain*.

[51] Bradshaw DH, Empy C, Davis P, Lipschitz D, Nakamura Y, Chapman CR (2008). Trends in funding for research on pain: a report on the national institutes of health grant awards over the years 2003 to 2007. *Journal of Pain*.

[52] Lynch ME, Schopflocher D, Taenzer P, Sinclair C (2009). Research funding for pain in Canada. *Pain Research and Management*.

